# Efficient Editing of the ZBED6-Binding Site in Intron 3 of IGF2 in a Bovine Model Using the CRISPR/Cas9 System

**DOI:** 10.3390/genes13071132

**Published:** 2022-06-24

**Authors:** Huiying Zou, Dawei Yu, Shun Yao, Fangrong Ding, Junliang Li, Ling Li, Xue Li, Shanjiang Zhao, Yunwei Pang, Haisheng Hao, Weihua Du, Xueming Zhao, Yunping Dai, Huabin Zhu

**Affiliations:** 1 Embryo Biotechnology and Reproduction Laboratory, Institute of Animal Sciences, Chinese Academy of Agricultural Sciences, Beijing 100193, China; zouhuiying@caas.cn (H.Z.); ydw023@163.com (D.Y.); yaoshun18877106195@163.com (S.Y.); lijunliangyx@163.com (J.L.); zhaoshanjiang@caas.cn (S.Z.); pangyunwei@caas.cn (Y.P.); haohaisheng@caas.cn (H.H.); duweihua@caas.cn (W.D.); zhaoxueming@caas.cn (X.Z.); 2 State Key Laboratory of Agrobiotechnology, College of Biological Sciences, China Agricultural University, Beijing 100193, China; dingfangrong@aliyun.com (F.D.); jaysionli@126.com (L.L.); lixue2018020010@163.com (X.L.)

**Keywords:** CRISPR/Cas9 system, gene editing, insulin-like growth factor 2, ZBED6-binding site, cattle

## Abstract

Background: Insulin-like growth factor 2 is a growth-promoting factor that plays an important role in the growth and development of mammals. A nucleotide substitution in intron 3 of *IGF2*—which disrupts the ZBED6-binding site—affects muscle mass, organ size, and fat deposition in pigs. The ZBED6-binding site is also conserved in cattle. Methods: In the present study, we introduced mutations in the ZBED6-binding site in intron3 of *IGF2* in bovine fetal fibroblasts using the CRISPR/Cas9 system, and investigated the effect of disruption of ZBED6 binding on *IGF2* expression. Results: Eleven biallelic-mutant single-cell clones were established, three of which contained no foreign DNA residues. Single-cell clones 93 and 135 were used to produce cloned embryos. Dual-luciferase reporter assay in C2C12 cells demonstrated that the mutation in the ZBED6-binding site increases the promoter 3 activity of bovine *IGF2*. A total of 49 mutant cloned embryos were transplanted into surrogate cows. Unfortunately, all cloned embryos died before birth. IGF2 was found to be hypomethylated in the only fetus born (stillborn), which may have been due to the incomplete reprogramming. Conclusions: We efficiently constructed IGF2-edited cell lines and cloned embryos, which provided a theoretical basis and experimental materials for beef cattle breeding.

## 1. Introduction

Insulin-like growth factor 2 (*IGF2*) is an important growth-promoting factor, which is mainly produced by the liver, and binds to target cell surface receptors to promote cell migration, proliferation, and differentiation [1]. *IGF2* plays a central role in embryonic development and muscle cell proliferation in livestock. *IGF2* expression is regulated by multiple promoters in mammals; for example, both human *IGF2* (P1–P4) and murine *Igf2* have four promoters [2], and murine *Igf2* includes three promoters (P1–P3) and an upstream promoter (P0) [3,4]. During human embryonic development, *IGF2* expression is mainly driven by the P2–P4 promoters, whereas after birth, *IGF2* expression gradually decreases. In adult tissues, *IGF2* expression is mainly regulated by the P1 promoter [5,6].

The role of *IGF2* in muscle growth regulation was first identified in pigs. A quantitative trait locus (QTL) associated with muscle growth, backfat thickness, and heart size was identified in a wild boar × Large White intercross, and the QTL was subsequently found to be located in an *IGF2* non-coding region (*IGF2*-intron3-3072) [7,8,9]. A G-to-A mutation in the QTL has been reported to result in a threefold increase in *IGF2* expression in muscles, a 3–4% increase in pig meat production, and a 20% reduction in backfat thickness [9]. *IGF2* expression is not different in fetal muscles, and the difference occurs mainly during piglets’ growth after birth. Zinc finger BED-type containing 6 (*ZBED6*) is a newly discovered transcription factor regulating muscle growth, and is conserved among placental mammals [10,11]. The G-to-A mutation or ZBED6-binding site deletion of *IGF2* disrupts the binding of the transcriptional repressor ZBED6, thereby increasing the expression of *IGF2* [12,13]. Both *Zbed6*-knockout mice and ZBED6-binding-site-mutant mice showed enlargement of muscles and internal organs, similar to the findings in pigs with corresponding mutations [14,15]. Furthermore, the muscle enlargement in mice was significantly greater than that in pigs (15–20% vs. 3–4%, respectively), probably because of the long-term selection of pigs for muscle growth [15]. Several studies have investigated the effects of mutations in the ZBED6-binding site in pigs created using CRISPR/Cas9 technology [16,17]. In one study, mutant pigs showed a significant increase in muscle mass, especially in the buttocks, shoulders, and legs. The body weight of 175-day-old and 338-day-old mutant pigs was 18% and 32% higher, respectively, than that of wild-type pigs [16]. In another study, Chinese Bama pigs with a ZBED6-binding-site mutation were obtained via microinjection using the CRISPR/Cas9 system. There was no difference in the birth weight of the mutant Bama pigs and wild-type pigs. However, the growth of knockout pigs was significantly faster than that of wild-type pigs, leading to significant differences in body size [17]. The above findings indicate that ZBED6–*IGF2* regulation plays an important role in regulating the growth of muscle and internal organs in placental mammals. Moreover, the sequence around the ZBED6-binding site in the *IGF2* gene shows high homology in placental mammals; thus, the mechanism of ZBED6–*IGF2* regulation of muscle growth is very conservative [18].

CRISPR/Cas9 technology, employing the Cas9 protein guided by sgRNA for gene editing, has been widely used for disease-resistance breeding and trait improvement in livestock [19,20,21,22]. The present study aimed to establish a ZBED6-binding site double-mutant bovine fibroblast cell line using CRISPR/Cas9 technology, and to explore the effect of ZBED6-binding-site deletion on *IGF2* promoter activity. Furthermore, mutant embryos were produced by somatic cell nuclear transfer (SCNT) to investigate the effect of ZBED6-binding-site deletion on the development of cloned embryos. Our study could provide novel resources and new methods for beef cattle breeding.

## 2. Materials and Methods

### 2.1. sgRNA Design and Verification

Five pairs of sgRNA were designed based on the ZBED6-binding site in intron 3 of the bovine *IGF2* gene using the CRISPR design tool (Zhang lab). The sequence of sgRNAs is displayed in Table S1. Annealed sgRNA was cloned into the pX458 vector (Addgene, Watertown, DC, USA) using BbsI. Bovine fetal fibroblasts (1 × 10^6^; derived from Angus cattle) were transfected with 3 µg of pX458-sgRNA according to the manufacturer’s instructions (Lonza, Basel, Switzerland). After 48–72 h, genomic DNA was extracted, and the target locus mutation was verified by T7 endonuclease I digestion and Sanger sequencing.

### 2.2. T7 Endonuclease I (T7E1) Digestion

T7E1 can recognize and cleave imperfectly matched DNA. Primers were designed on the basis of sequences upstream and downstream of sgRNA target sites. Approximately 400 ng of PCR products, 1 µL of 10 × Taq DNA polymerase buffer (Takara, Dalian, China), and ddH_2_O, in a final volume of 10 µL, were used to conduct denaturation and slow annealing. Next, 1.5 µL of T7E1 reaction buffer (NEB, Ipswich, MA, USA), 3 µL of ddH_2_O, and 0.5 µL of T7E1 (NEB, Ipswich, MA, USA) were added to the annealed product at a final volume of 15 µL, and the reaction mixture was incubated for 1 h at 37 °C. The digestion products were detected by electrophoresis using an 8% polyacrylamide gel or 2.5% agarose gel. The intensity of the digested and undigested bands was analyzed using ImageJ software to calculate the mutation efficiency of the five sgRNAs.

### 2.3. TA Cloning

PCR products of sgRNA target sites were ligated to the PMD-19T vector (Takara, Dalian, China) and incubated for 2–4 h at 16 °C. The digestion products were transfected into DH5a-competent cells (TransGen, Beijing, China). Bacterial colonies were chosen for PCR. Bacterial colonies containing correctly inserted DNA fragments were sent for sequencing. The sequencing results were aligned with the wild-type sequence using the alignment tool of DNAMAN software to analyze mutation types and calculate mutation efficiency.

### 2.4. Dual-Luciferase Reporter Assay

The predicted P3 promoter of bovine *IGF2* was amplified from bovine fetal fibroblasts and cloned into a HindIII-digested pGL3 basic vector (Promega, Madison, WI, USA) to obtain the pGL3-P3 vector. Fragments of 578 bp containing the mutation site were amplified from bovine fetal fibroblast cell lines containing a homozygous mutation and cloned into KpnI/NheI-digested pGL3-P3 vector with KpnI/NheI to obtain the pGL3-P3-93 vector. C2C12 cells (5 × 10^5^) were transfected with 2 µg of pGL3-P3-93 and 200 ng of pRL-TK. After 36–48 h, cells were collected to detect the luciferase activity according to the protocol of the Dual-Luciferase Assay System (Promega, Madison, WI, USA).

### 2.5. Single-Cell Clone Culture and Identification

Bovine fetal fibroblasts (1 × 10^6^) were transfected with the vector pX458-sgRNA1 (6 µg), as it showed the highest mutation efficiency. After 24 h, we used flow-cytometry-based cell sorting to obtain cells with GFP expression from the pX458-sgRNA1 vector. Then, the cells were seeded into 10 cm dishes with 150–200 cells per dish. Single-cell clones were selected for expansion and propagation. Until the cells reached >90% confluency, a fraction of the cells was used for genome extraction. PCR was conducted using primers targeting the sequences around the sgRNA target sites. The PCR products were sequenced to verify the target site mutations. Homozygous mutant cell clones were used for SCNT to acquire cloned embryos.

### 2.6. Somatic Cell Nuclear Transfer

Mature oocytes were enucleated by micromanipulation using a pipette with an inner diameter of 20 µm. Donor cells were injected into the perivitelline space of the enucleated oocytes. Reconstructed embryos were placed into the incubator for 1 h. Then, the reconstructed embryos were activated to fuse using an electrofusion instrument with a pulse (2.2 kV/cm, 20 µs). The reconstructed embryos were incubated in a culture medium supplemented with ionomycin for 5 min, and then washed three times with PBS. The reconstructed embryos were transferred to a culture medium supplemented with 6-dimethylaminopurine (6-DMAP) for 6–8 h. Finally, the successfully fused embryos were selected and placed in mineral-oil-covered medium droplets with 15–20 embryos per droplet, and observed under a stereomicroscope.

### 2.7. Bisulfite Genomic Sequencing

Genomic DNA was extracted from bovine ears following the protocol of the TIANamp Genomic DNA Kit (TIANGEN, Beijing, China). Approximately 100–500 ng of genomic DNA was subjected to bisulfite treatment using the MethylDetector Bisulfite Modification Kit (Active Motif, Carlsbad, CA, USA) according to the manufacturer’s protocol. The treated DNA was amplified using bisulfite sequencing primers. The PCR products were ligated to the pMD19-T vector. At least 10 bacterial colonies were sequenced. The sequencing results were analyzed using a quantification tool (QUMA; http://quma.cdb.riken.jp/ accessed on 7 March 2022).

## 3. Results

### 3.1. Identification of the ZBED6-Binding Site in Bovine IGF2 Intron 3 by Homologous Alignment

**Figure 1:**
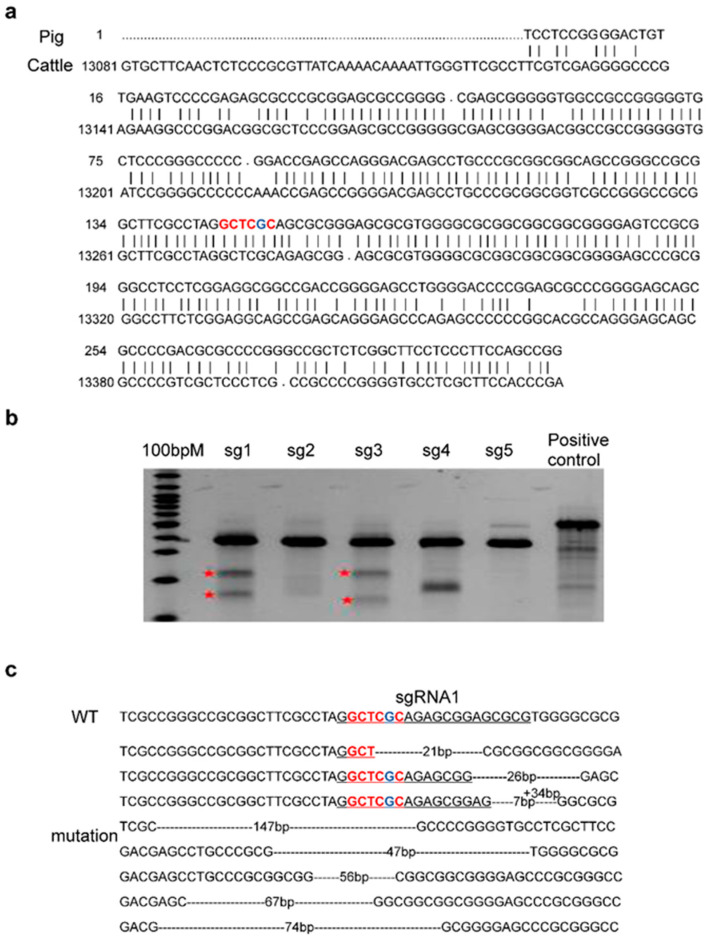
Prediction of the ZBED6-binding site in bovine *IGF2*, and determination of sgRNA mutation efficiency: (**a**) Identification of the ZBED6-binding site in bovine *IGF2* intron 3 using homologous alignment. The upper bases of each line are the porcine IGF2 sequence; the lower bases of each line are the bovine *IGF2* sequence. (**b**) Determination of the mutation efficiency of five pairs of sgRNA by T7 endonuclease 1 digestion. The red asterisk marks the fragments digested by T7 endonuclease 1. (**c**) Determination of the mutation efficiency of sgRNA1 using TA cloning. Bases in red represent the ZBED6-binding site, the base in blue represents the key base of the ZBED6-binding site, underlined bases indicate sgRNA, and dashed lines represent base deletion.

Because there are few studies on the bovine *IGF2* gene, we first identified the ZBED6-binding site in the *IGF2* intron by homologous alignment. The 300 bp sequence around the ZBED6-binding site (5′-GCTCGC-3′) in intron 3 of porcine *IGF2* was aligned with the bovine *IGF2* gene (Accession number: NC_037356.1), and a homologous sequence in bovine IGF2 intron 3 with 84% homology with the porcine sequence was identified. The homologous sequence includes the ZBED6-binding site, and the important QTL is located at the bovine IGF2 intron 3 nucleotide 3104 (Figure 1a).

### 3.2. Design of IGF2 sgRNAs and Mutation Efficiency Verification

Five pairs of sgRNA were designed based on the ZBED6-binding site in the *IGF2* intron 3. Bovine fetal fibroblasts were transfected with the sequenced pX458-sgRNA plasmids. The PCR product amplified from the genomic DNA of bovine fetal fibroblasts was digested with T7E1 to evaluate the mutation efficiency. Among the five pairs of sgRNA, sgRNA1 had the highest mutation efficiency, at approximately 27% (Figure 1b). The mutation efficiency of sgRNA1 was further analyzed using TA clone sequencing of its PCR product; 8 of the 32 bacterial colonies showed insertion, deletion, and replacement mutations, with an efficiency of 25% (Figure 1c), which was consistent with the results obtained from T7E1 digestion. SgRNA1 was used for subsequent experiments.

### 3.3. Construction of IGF2 Mutant Cell Lines with no Foreign DNA Residues

**Figure 2:**
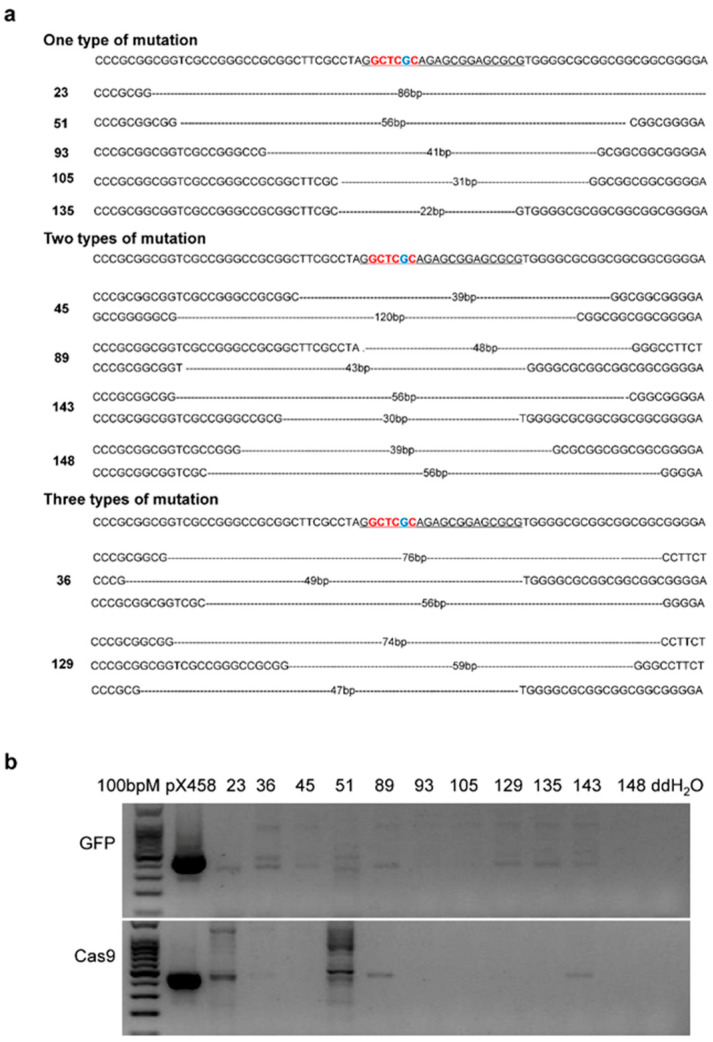
Identification of mutation types and detection of foreign DNA residues in homozygous mutant single-cell clones: (**a**) Identification of mutation types in homozygous mutant single-cell clones via TA clone sequencing. Bases in red represent the ZBED6-binding site, the base in blue represents the key base of the ZBED6-binding site, underlined bases indicate sgRNA, and dashed lines represent base deletion. (**b**) Detection of foreign DNA residues in homozygous mutant single-cell clones using PCR. The upper image shows the fragments amplified from homozygous mutant single-cell clones using primers designed based on the *GFP* gene; the lower image shows the fragments amplified from homozygous mutant single-cell clones using primers designed based on the *Cas9* gene.

Bovine fetal fibroblasts were transfected with the pX458-sgRNA1 plasmid, and 159 single-cell clones were obtained via flow-cytometry-based cell sorting and dilution culture. PCR and sequencing were performed to detect sgRNA1 target site mutations. Of the 159 single-cell clones, 141 were successfully sequenced. We analyzed the sequencing results and found that 89 single-cell clones showed monoallelic or biallelic mutations. Eleven (23, 36, 45, 51, 89, 93, 105, 129, 135, 143, and 148) single-cell clones were shown to have biallelic deletion of the ZBED6-binding site, as verified by TA cloning and sequencing (Figure 2a). Five clones had one type of mutation, four clones had two types of mutation, and two clones had three types of mutation. The homozygous mutation efficiency was approximately 7.8%. In order to detect the presence of foreign DNA residues, we performed PCR on the *Cas9* and green fluorescent protein (*GFP*) genes of the pX458-sgRNA plasmid, and found that three single-cell clones (93, 105, and 148) had no foreign DNA residues (Figure 2b).

### 3.4. ZBED6-Binding-Site Mutation Can Increase the Expression of IGF2

**Figure 3:**
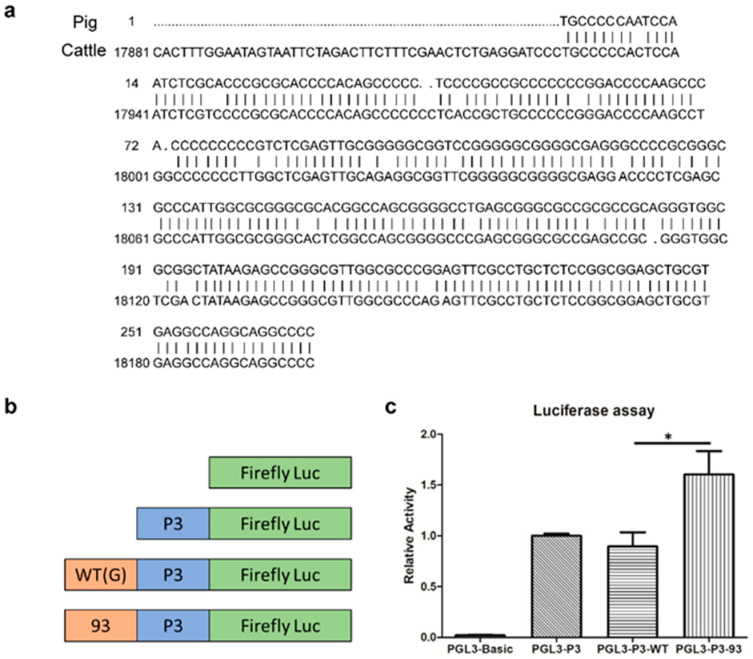
Analysis of the effect of ZBED6-binding site knockout on *IGF2* P3 activity in murine C2C12 cells, by dual-luciferase reporter assay: (**a**) Identification of the bovine *IGF2* P3 promoter using homologous alignment. The upper bases of each line are the porcine *IGF2* P3 sequence; the lower bases of each line are the bovine *IGF2* sequence. (**b**) Design of the four experimental plasmids. The orange rectangle refers to the 578 bp fragment containing the ZBED6-binding site, the blue rectangle refers to the *IGF2* P3 promoter, and the green rectangle refers to the luciferase gene. (**c**) Relative luciferase expression in cells transfected with different plasmids. Data are presented as the mean ± SD (*n* = 3); * indicates *p* ≤ 0.05.

To investigate whether ZBED6-binding site deletion can increase *IGF2* expression, we performed a dual-luciferase reporter assay in C2C12 murine myoblast cells. First, bovine *IGF2* P3 promoter was predicted by homologous alignment. The porcine *IGF2* P3 promoter was aligned with the bovine *IGF2* gene (Accession number NC_037356.1). A homologous sequence (with homology of 90%) was found in bovine *IGF2*, which was identified as the bovine *IGF2* P3 promoter (Figure 3a). Then, we examined the effect of ZBED6-binding-site deletion on P3 promoter activity in C2C12 cells using a dual-luciferase assay. The P3 promoter fragment was ligated into a HindIII-digested pGL3 basic vector to construct the pGL3-P3 plasmid. Fragments (578 bp) flanking the ZBED6-binding site amplified from the wild-type bovine genome and the genome of the single-cell 93 clone were ligated into KpnI- and NheI-digested pGL3-P3 vectors to construct pGL3-P3-WT and pGL3-P3-93, respectively (Figure 3b). C2C12 cells were co-transfected with the control plasmid pRL-TK and the pGL3 basic, pGL3-P3, pGL3-P3-WT, or pGL3-P3-93 plasmids. Cells were harvested after 24–48 h to determine luciferase expression. The expression level of luciferase in cells transfected with the pGL3-P3-93 plasmid containing a ZBED6-binding-site deletion was significantly higher (*p* ≤ 0.05, approximately 1.8 times) than that in cells containing the pGL3-P3-WT plasmid (Figure 3c).

### 3.5. Production of IGF2-Mutant Embryos by Somatic Cell Nuclear Transfer

**Figure 4:**
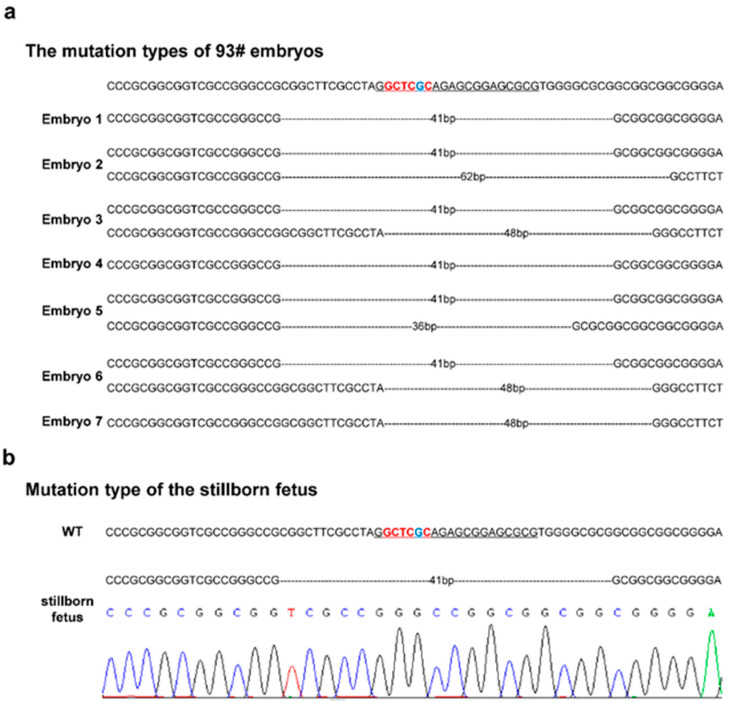
Mutation types in 93 cloned bovine embryos: Identification of mutation types using TA clone sequencing (**a**) in embryos cloned using the single-cell clone 93, and (**b**) in a stillborn fetus. Bases in red represent the ZBED6-binding site, the base in blue represents the key base of the ZBED6-binding site, underlined bases indicate sgRNA, and dashed lines represent base deletion.

The single-cell clones 93 and 135 were used to produce cloned embryos by SCNT (Table 1). In order to evaluate the homogeneity of single-cell clones, 7 embryos cloned using single-cell clone 93 and 10 embryos cloned using single-cell clone 135 were sequenced by TA cloning. For cloned embryos, the main mutation types were 41 bp deletion and 22 bp deletion, which corresponded with that in single-cell clones 93 and 135, respectively. Some embryos showed different types of mutations, which may have been due to the limited number of bacterial colonies sequenced for single-cell clones. All embryos showed ZBED6-binding-site deletion, and no wild type was detected (Figure 4a, Figure S1). Therefore, embryos cloned using the single-cell clones 93 and 135 can be used for embryo transfer to produce cloned cattle with ZBED6-binding-site mutation. In addition, Cas-OFFinder software was used to evaluate the off-target effects induced by the CRISPR/Cas9 system, and four potential off-target sites (OT1–OT4) were detected (Table S2). Sequences around off-target sites were amplified by PCR from genomic DNA of single-cell clones 93 and 135. The results of Sanger sequencing showed that no off-target editing occurred in single-cell clones used for producing cloned embryos, indicating the accuracy of the CRISPR/Cas9 system (Figure S2).

### 3.6. IGF2 Methylation in IGF2-Mutant Cloned Embryos

**Figure 5:**
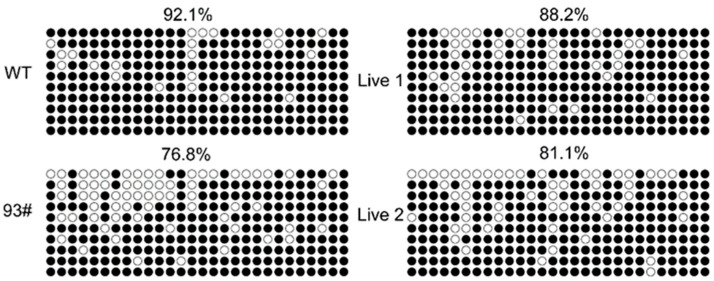
*IGF2* methylation status in stillborn cloned cattle: Methylation levels of *IGF2* DMR in wild-type cattle, stillborn cloned cattle, and live cloned cattle. WT represents wild-type cattle; 93# represents stillborn cloned cattle; Live 1 and Live 2 represent live cloned cattle. Each circle represents CpG dinucleotides. The methylation level (%) was based on the percentage of methylated CpGs in all examined CpGs; open circles represent no methylation and filled circles represent methylation.

In total, 17 embryos cloned using single-cell clone 93 were transplanted into the uteri of 17 recipient cows; moreover, 32 embryos cloned using single-cell clone 135 were transplanted into 32 recipient cows (Table 2). Unfortunately, the transplantation of most of the cloned embryos resulted in miscarriage, whereas one embryo resulted in stillbirth; an ear sample was taken from the stillborn fetus for PCR and sequencing, which identified it as a 93-cloned embryo (Figure 4b). All 15 TA clones of the stillborn fetus showed a 41 bp deletion. Methylation analysis showed that the *IGF2* differentially methylated region (DMR) of the 93-cloned embryo was hypomethylated compared with living cloned cattle and wild-type cattle (Figure 5). We speculate that aberrant methylation of the *IGF2* DMR may be one of the reasons for the death of cloned cattle. In addition, the results of the off-target effect detection of cloned cattle showed that there was no mutation at the potential off-target sites (Figure S2).

## 4. Discussion

*ZBED6* is an important transcription factor that regulates *IGF2* expression by binding a conserved motif (GCTCG) [11,23]. Knockout of the ZBED6-binding site can enhance *IGF2* expression at the cellular level and increase the proliferative ability of muscle cells. The ZBED6-binding site in *IGF2* intron 3 is ideal for gene editing because deleting the ZBED6-binding site does not destroy the coding region of *IGF2*, and does not affect the characteristics of the IGF2 protein. Knockout experiments in pigs and mice have shown that knocking out the ZBED6-binding site can increase *IGF2* expression, thereby significantly increasing the growth rate of mice and pigs [7,16,17]. ZBED6-binding-site knockout has higher biosafety than *MSTN* knockout, and the ZBED6-binding site is an economically valuable region in terms of gene editing. Our study also supports the suppression of *IGF2* expression by ZBED6 binding in cattle [24]. Results from the dual-luciferase reporter assay showed that mutation of the bovine ZBED6-binding site significantly upregulated luciferase expression under the bovine *IGF2* P3 promoter, indicating that ZBED6-binding-site deletion can enhance IGF2 P3 promoter activity, and that the conserved inhibitory effect of ZBED6 on *IGF2* expression also exists in cattle.

In our study, single-cell clones were acquired via flow-cytometry-based cell sorting on the basis of the presence of GFP, and no drug resistance pressure was used during the selection and culturing processes. Most of the sgRNA vectors were lost during cell proliferation. We also used PCR to identify the residual state of foreign vectors, and acquired single-cell clones without foreign gene residues, which made our new breeding materials more biologically safe. Of the 141 successfully sequenced single-cell clones, 89 were mutant clones, of which 78 and 11 showed monoallelic and biallelic mutations, respectively. Thus, flow-cytometry-based cell sorting is a very effective screening method. The mutation patterns observed in the homozygous cell clones were diverse, including one, two, and three types of mutations. In addition to the main mutations (the 41 bp deletion and 22 bp deletion), the seven embryos cloned using single-cell clone 93 contained many other mutation patterns, which may have been due to the low sequencing depth of TA clone sequencing, meaning that a small proportion of the mutation patterns in the single-cell clones could not be identified. The multiple mutation patterns in the single-cell clones and cloned embryos indicated that sgRNA vectors may persist for more than one cell cycle in bovine fetal fibroblasts.

For the SCNT, apart from single-cell clone 93—which showed no foreign gene residues—we also selected single-cell clone 135, with the smallest deletion. However, all 49 cloned embryos died after transplantation. The sole stillbirth showed hypomethylation in the *IGF2* DMR, which may have been due to the incomplete reprogramming of the cloned embryos. Improper reprogramming of epigenetic modifications during SCNT can cause embryonic defects. These reprogramming errors include imprinted genes, histone modifications, and X-chromosome inactivation [25]. The survival rate of cloned bovine embryos is about 5–10% [26]. The majority of cloned embryos died because of the cloning technology. Nevertheless, we cannot rule out the possibility that the deaths were caused by high *IGF2* expression. The *IGF2* gene is a paternally imprinted gene that is associated with fetal growth [27]. Abnormal methylation of the *IGF2* DMR and ectopic *IGF2* expression in cloned embryos can cause symptoms such as large offspring and placental defects [28,29,30]. Although ZBED6-binding-site knockout in mice and pigs did not eliminate the imprinting effect of *IGF2*, the inhibitory effect of ZBED6 on *IGF2* needs to be further studied in cattle. In addition, in order to avoid the embryonic defects caused by SCNT, we intend to use pronuclear injection with the CRISPR/Cas9 system in embryos fertilized in vitro to explore the effects of eliminating the inhibitory effect of ZBED6 on *IGF2* on bovine development.

## 5. Conclusions

We successfully constructed ZBED6-binding-site-knockout cell lines and cloned embryos, with no foreign gene residues. Furthermore, the results of dual-luciferase reporter assay in C2C12 cells showed that knocking out the ZBED6-binding site can increase the activity of the bovine *IGF2* promoter, indicating that ZBED6–*IGF2* regulation is conserved in cattle. However, due to the high failure rate inherent to somatic cell cloning, we did not obtain live *IGF2*-edited cattle. In the future, more research is needed to investigate the regulation of *IGF2* and obtain high-yield beef cattle by editing *IGF2*.

## Figures and Tables

**Table 1:** In vitro development of NT embryos derived from single-cell clones 93 and 135.

Donor Cells	No. of Oocytes	No. of Reconstructed Embryos	No. of Blastocysts	Blastocysts Rate %
93	240	183	81	44.3
135	487	366	116	31.7
WT	551	464	176	31.9

**Table 2:** In vivo development of NT embryos derived from single-cell clones 93 and 135.

Donor Cells	No. of Transferred Embryos	No. of Recipients	No. of Pregnancies GD60	No. of Live Cloned Cattle
93	17	17	2	0
135	32	32	5	0
WT	41	41	8	2

Note: one cloned cow from single-cell clone 93 was stillborn.

## Data Availability

Not applicable.

## Supplementary Materials

The following supporting information can be downloaded at: https://www.mdpi.com/article/10.3390/genes13071132/s1, Table S1: List of primers used in this study. Table S2: Potential off-target sites. Figure S1: Mutation types in 135 cloned bovine embryos. Figure S2: Detection of the mutation at potential off-target sites by Sanger sequencing.
