# Supplementary material for: Efficient Editing of the ZBED6-Binding Site in Intron 3 of IGF2 in a Bovine Model Using the CRISPR/Cas9 System
Source: Genes (Basel). 2022 Jun 24;13(7):1132. doi: 10.3390/genes13071132 (PMC9325003; doi:10.3390/genes13071132)
Supplement: Supplementary file 1 [file genes-13-01132-s001.zip › Figure S1.pdf]

Supplementary Figure S1

## The mutation types of 135# embryos

CCCGCGGCGGTCGCCGGGCCGCGGCTTCGCCTAGGCTCGCAGAGCGGAGCGCGTGGGGCGCGGCGGCGGCGGGGA

**Embryo 1**
CCCGCGGCGGTCGCCGGGCCGCGGCTTCGC--------------------22bp------------------GTGGGGCGCGGCGGCGGCGGGGA
CCCGCGGCGGTCGCCGGGCCG-------------------------------------41bp----------------------------------------GCGGCGGCGGGGA

**Embryo 2** CCCGCGGCGGTCGCCGGGCCGCGGCTTCGC--------------------22bp-----------------GTGGGGCGCGGCGGCGGCGGGGA

**Embryo 3** CCCGCGGCGGTCGCCGGGCCGCGGCTTCGC--------------------22bp-----------------GTGGGGCGCGGCGGCGGCGGGGA

**Embryo 4** CCCGCGGCGGTCGCCGGGCCGCGGCTTCGC--------------------22bp-----------------GTGGGGCGCGGCGGCGGCGGGGA

**Embryo 5**
CCCGCGGCGGTCGCCGGGCCGCGGCTTCGC--------------------22bp------------------GTGGGGCGCGGCGGCGGCGGGGA
CCCGCGGCGGTCGCCGGGCCG-------------------------------------41bp----------------------------------------GCGGCGGCGGGGA

**Embryo 6**
CCCGCGGCGGTCGCCGGGCCGCGGCTTCGC--------------------22bp-----------------GTGGGGCGCGGCGGCGGCGGGGA
CCCGCGGCGGTCGC-------------------------------------------------------56bp-------------------------------------------------------GGGGA

**Embryo 7**
CCCGCGGCGGTCGCCGGGCCGCGGCTTCGC--------------------22bp-----------------GTGGGGCGCGGCGGCGGCGGGGA
CCCGCGGCGGT-----------------------------------------43bp----------------------------------------GGGGCGCGGCGGCGGCGGGGA

**Embryo 8**
CCCGCGGCGGTCGCCGGGCCGCGGCTTCGC--------------------22bp------------------GTGGGGCGCGGCGGCGGCGGGGA
CCCGCGGCGGTCGCCGGGCCG-------------------------------------41bp----------------------------------------GCGGCGGCGGGGA

**Embryo 9**
CCCGCGGCGGTCGCCGGGCCGCGGCTTCGC--------------------22bp------------------GTGGGGCGCGGCGGCGGCGGGGA
CCCGCGGCGGTCGCCGGGCCGGCGGCTTCGCCTA------------------------------48bp------------------------------GGGCCTTCT

**Embryo 10** CCCGCGGCGGTCGCCGGGCCGCGGCTTCGC--------------------22bp------------------GTGGGGCGCGGCGGCGGCGGGGA
