# Supplementary material for: Efficient Editing of the ZBED6-Binding Site in Intron 3 of IGF2 in a Bovine Model Using the CRISPR/Cas9 System
Source: Genes (Basel). 2022 Jun 24;13(7):1132. doi: 10.3390/genes13071132 (PMC9325003; doi:10.3390/genes13071132)

Supplementary Figure S2

Figure S2 Detection of the mutation at potential off-target sites by Sanger sequencing.

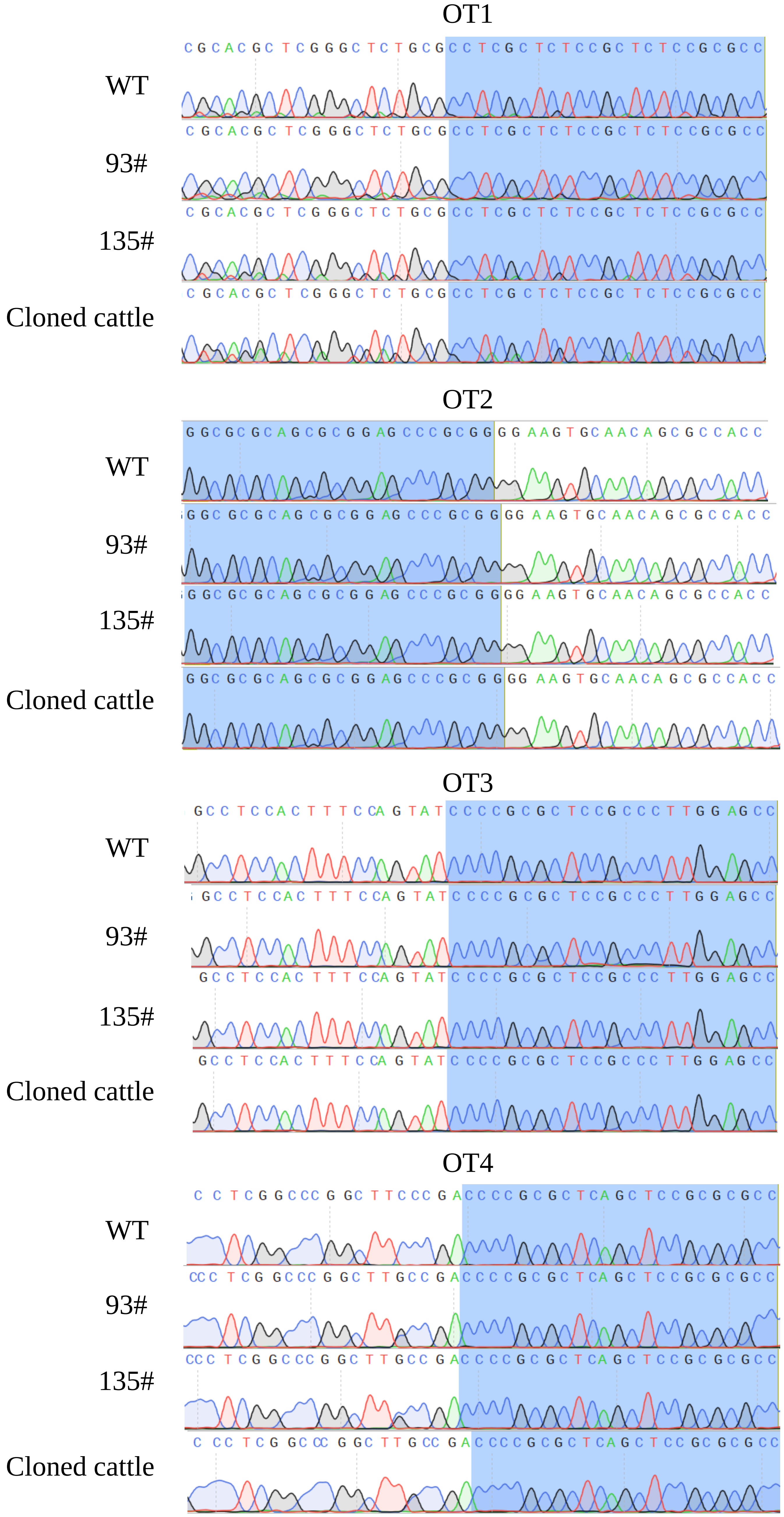

Supplement: Supplementary file 1 [file genes-13-01132-s001.zip › Figure S2.pdf]
