# Supplementary material for: Efficient Editing of the ZBED6-Binding Site in Intron 3 of IGF2 in a Bovine Model Using the CRISPR/Cas9 System
Source: Genes (Basel). 2022 Jun 24;13(7):1132. doi: 10.3390/genes13071132 (PMC9325003; doi:10.3390/genes13071132)
Supplement: Supplementary file 1 [file genes-13-01132-s001.zip › Table S1.pdf]

Supplementary Table S1

List of primers used in this study.

primer	sequence	
IGF2sgRNA1	ggctcgcagagcggagcgcg	SgRNAs for IGF2 and primers for amplification of sgRNA target sites
IGF2sgRNA2	gccgcgggcaggctcgtccc	
IGF2sgRNA3	agagcggagcgcgtggggcg	
IGF2sgRNA4	gcctaggcgaagccgcggcc	
IGF2sgRNA5	gcctgcccgcggcggtcgcc	
IGF2-F/R	cgccttctcttcaccagtgctt<br>gcttccgtgctgcagcgcgtc	
pXGFP-F/R	cggccacaagttcagcgtgtc<br>gcacgctgccgtcctcgatg	Amplification of DNA residue from pX458 vector
pXCas9-F/R	aagatatcgtgctgaccctga<br>atttcgatcacgatgttctcg	
HindIII P3-F/R	cccaagcttcccccactccaatctcgtccccg<br>cccaagcttggggcctgcctggcctcacgcag	Primers used for luciferase activity analysis
KpnI 578-F	ggggtacccttcaccagtgcttcaactctcc	
NheI 578-R	ctagctagcaagcaccgcgccgctgcggatcc	
OT1-F/R	ggccatccacatcccgcagac<br>gagccagggaaggtagaggagc	Primers used for off-target effects analysis
OT2-F/R	cgcataagccgaacaggtcgatc<br>cggtgggacagtcaagtccctc	
OT3-F/R	cgacccaggcctaccctccaag<br>ggtcacccaaatctccacttctc	
OT4-F/R	tcgacgtcactgatgcgtcacc<br>cctgtgaatctacaacgccttg	
IGF2DMR1F/1R	taatatgatatttggaagtagt<br>acatttttaaaaatattattct	Primers used for IGF2 DMR methylation
