# Supplementary material for: Efficient Editing of the ZBED6-Binding Site in Intron 3 of IGF2 in a Bovine Model Using the CRISPR/Cas9 System
Source: Genes (Basel). 2022 Jun 24;13(7):1132. doi: 10.3390/genes13071132 (PMC9325003; doi:10.3390/genes13071132)
Supplement: Supplementary file 1 [file genes-13-01132-s001.zip › Table S2.pdf]

Supplementary Table S2

Potential off-target sites.

Off-target	Sequence	Chromosome	Position	Direction
OT1	ggcgcggagagcggagagcgagg	chr7	19230399	-
OT2	ggcgcgcagcgcggagcccgcgg	chr21	7073571	-
OT3	ggctccaagggcggagcgcgggg	chr26	22462098	+
OT4	ggcgcgcggagctgagcgcgggg	chr28	6848215	-
